# Teamwork makes the dream work: IPA1 and OsNPR1 work together to enhance immunity in rice

**DOI:** 10.1093/plcell/koag153

**Published:** 2026-05-29

**Authors:** Annabelle Campbell

**Affiliations:** Assistant Features Editor, The Plant Cell, American Society of Plant Biologists; Department of Biology, Duke University, Durham, NC 27710, United States

When plant cells detect bacterial pathogens during infection, they elicit an immune response involving the biosynthesis and dissemination of the signaling hormone salicylic acid (SA). In Arabidopsis (*Arabidopsis thaliana)*, SA signaling is mediated by the protein NONEXPRESSOR OF PATHOGENESIS-RELATED GENES 1 (NPR1), a cytosolic-resident protein that translocates to the nucleus upon pathogen infection to interact with transcription factors that regulate defense-related genes (Shah et al. 1997; Kinkema et al. 2000). Within the nucleus, NPR1 turnover is mediated by the E3 ubiquitin ligase Cullin3 (CUL3) (Zavaliev et al. 2020). Like Arabidopsis NPR1, its ortholog in the rice plant *Oryza sativa*, OsNPR1, contributes to disease resistance and is degraded by OsCUL3a (rice ortholog of CUL3) (Liu et al. 2017). However, whether OsNPR1 also acts as a co-transcription factor for defense-related genes has been yet unknown.

Another rice protein of interest in the context of plant immunity is the transcription factor IDEAL PLANT ARCHITECTURE 1 (IPA1). IPA1 modulates expression of genes that regulate reactive oxygen species (ROS), abscisic acid levels, abiotic stress responses, and, namely, resistance to blast disease and bacterial blight (Wang et al. 2018; Liu et al. 2019). Like with OsNPR1, it was unknown which defense-related genes IPA1 regulates and how this activity is modulated. In recent work, Zhen Wang and colleagues (Wang et al. 2026) speculate that OsNPR1 and IPA1 interact to co-regulate immune activation in response to disease.

The authors first observed the phenotypes of plants overexpressing IPA1 (*IPA1*-OE) and plants overexpressing OsNPR1 (*OsNPR1*-OE). They noted that the 2 genotypes have shared characteristics indicative of a hyperactive immune system (Fig. 1, A–F), suggesting that the 2 proteins may act in the same pathway. Indeed, multiple types of binding assays show that IPA1 and OsNPR1 directly interact. Interestingly, application of an SA analog enhanced this binding interaction, and the *IPA1*-OE plants boosted SA signaling, suggesting a key role for these proteins in the SA signaling pathway.

**Figure 1:**
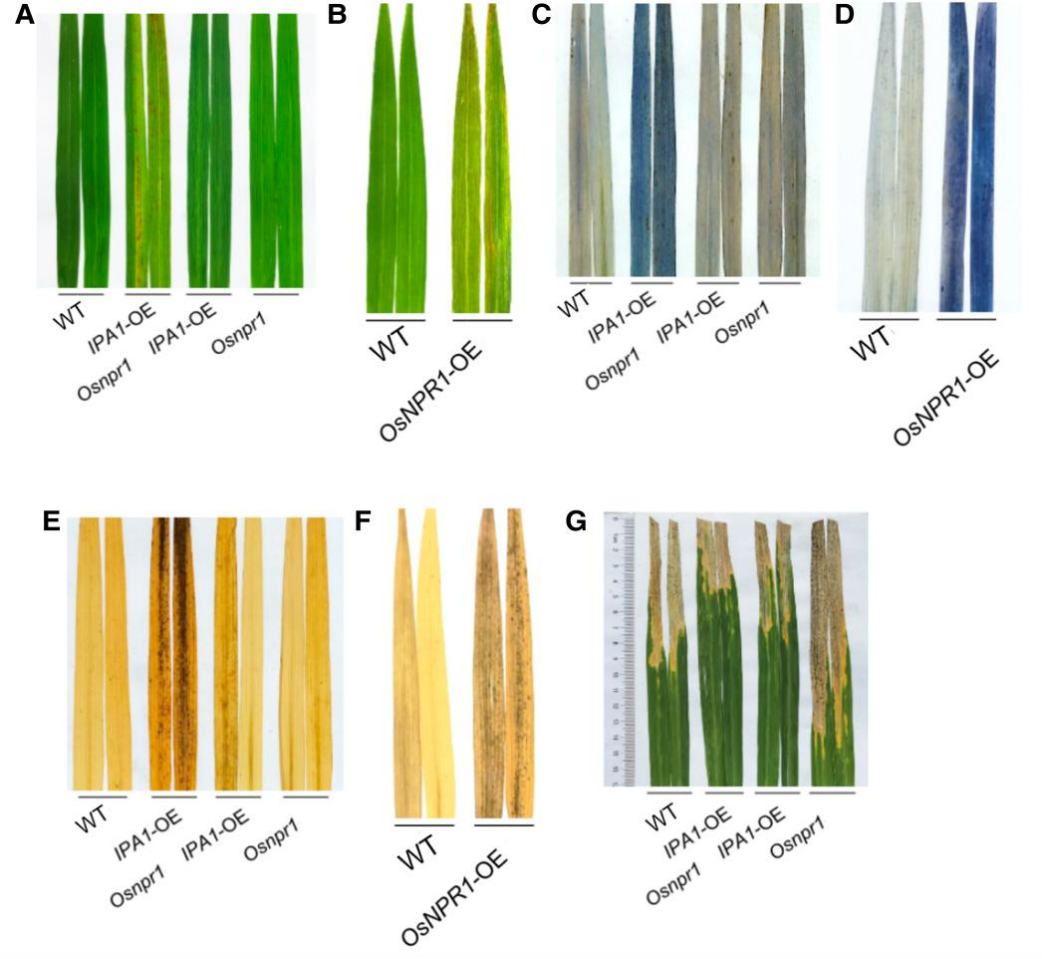
Hyperactive immune response in OsNPR1 and IPA1 overexpression genotypes. A & B) Plant lesions. C & D) Trypan blue staining for cell death. E & F) DAB staining for ROS accumulation. G) Lesions caused by bacterial blight infection. Adapted from Wang et al. (2026) Figure 2 and Figure S1.

To determine whether OsNPR1 is required for IPA1 function, *IPA1*-OE plants with an *Osnpr1* mutation were generated. Without *OsNPR1* expression, the plants no longer showed autoimmune/hyperactive immunity phenotypes associated with *IPA1* overexpression, indicating that OsNPR1 is required for IPA1's function in disease resistance (Fig. 1, A, C, E, and G).

With both OsNPR1 and IPA1 being transcription or co-transcription factors, the authors wanted to investigate the transcriptional profiles of each of the proteins and look for any overlap. RNA sequencing revealed that OsNPR1 and IPA1 largely regulate the expression of a common set of genes, and that OsNPR1 is required for IPA1-mediated regulation expression for many. To further investigate how OsNPR1 affects IPA1 activity, the authors used chromatin immunoprecipitation with quantitative PCR (ChIP-qPCR) and in vitro binding assays to reveal that IPA1 shows reduced binding to its defense-related gene targets in the absence of OsNPR1. These data, together with the OsNPR1-directed changes in *IPA1*-OE hyperactive immune phenotypes, support the hypothesis that OsNPR1 interacts with IPA1 to promote target gene binding and disease resistance.

Because targeted degradation of *Arabidopsis* NPR1 by CUL3 occurs in the nucleus, the authors of this study were motivated to investigate OsNPR1 stability. They confirmed previous observations that the E3 ligase OsCUL3a (ortholog to CUL3) mediates OsNPR1 turnover in the nucleus. Interestingly, they also found that IPA1 stabilizes nuclear-localized OsNPR1, likely by direct binding and outcompeting degradation machinery. The authors point out that posttranslational modifications of IPA1 drive its specific transcriptional activities, and that these or other modifications could possibly modulate its binding to OsNPR1.

In conclusion, this study provides evidence for a novel regulatory interaction between OsNPR1 and IPA1 to drive gene expression changes important for pathogen defense in rice, and that this interaction both contributes to and is driven by cellular SA levels. Research of this nature untangling the complex web of plant immunity, especially in crops, is an important contribution for efforts to engineer more resilient plants in response to a growing population and changing climate.

## Recent related articles in *The Plant Cell:*

- Tang et al. (2025) provide a review article investigating how plants have evolved to use disease tolerance over disease resistance to mitigate disease-induced damage, and how these insights can inform the development of disease tolerant crops.
- Sun et al. (2024) show that alternative splicing of the potato resistance gene *RB* balances plant growth and immunity, and the pathogen effector IPI-O1 interacts with the spliceosome to mediate the *RB* splicing and activate immunity.
- Zhong et al. (2026) demonstrate that the transcription factor MYC2 is phosphor-regulated by the phosphatase PP2A Bɑ and the kinase EDR1 to mediate powdery mildew resistance.

## Data Availability

The data underlying this article are available at https://doi.org/10.1093/plcell/koag122.
